# The Night is Dark and Full of Itches

**DOI:** 10.4269/ajtmh.21-0206

**Published:** 2021-06-15

**Authors:** Kaliaperumal Karthikeyan, M Prarthana

**Affiliations:** Department of Dermatology, Venereology & Leprosy, Sri Manakula Vinayakar Medical College & Hospital, Puducherry, India

“An itch, easy to scratch and when there is a reason, we celebrate.” Who can understand an itch better than a dermatologist, for it is their bread and butter.

I happened to meet a lady in my practice whose predominant complaint was itching. Her appearance gave an impression that she was impoverished and seemed to be in distress. When she came in, she immediately clicked on and started complaining about her issues. She had intractable pruritis due to scabies, which was not a surprise considering her state of affairs. She was naturally talkative and had an aura that made her get people around her conversational. She revealed that she stayed alone in a small scrambled up house with no company and worked as a day laborer to meet her needs. After all her complaints she ended up saying, “Saaar, neither can I work nor sleep with this itch and if you cannot treat me, please give me something to end my miserable life. My nights have become a nightmare which I can’t explain in words. I am a destitute and I do not have any relatives nor a reason to live. If my destiny is to suffer, then I would like to end it.” These were her words and in my honest opinion no one is a destitute in this world. The belief in destiny is deeply rooted in this part of the world, and she was no different.

One’s psychological and sentimental views go astray when poverty and sickness coexist. There are multiple scientific reasons as to why the mites itch at night. What the mite doesn’t know is that it targets the impoverished whose only pleasure is a night’s peaceful sleep. That sleep is necessary for their next day’s venture, existence, and food. In front of me was a lady who was ready to end her life to cease the itch. I treated her with appropriate medications and advised her to return back after 2 weeks and decided against charging her for the consult. She came to me a little furious demanding as to why I did not charge her. I didn’t have the courage to state the obvious and was beating around the bush. She hushed me with a determined voice. “Sir, I’m not a beggar. I make enough money to support myself. This isn’t professional ethics, and you need to be paid for your work.”

Oh my! I wasn’t expecting this. I have seen so many people use innumerable reasons to avoid paying the doctor’s fee. But she was one of a kind. With that note, she left a few currencies on my table and left.

Her story reflects the life of people from the developing world. In a country like India, people only dream of making their life meaningful, but in reality they do not have the means to and have to fight multiple battles to earn a livelihood. Health-related issues are a major hurdle and take up a major chunk of people’s time and energy on a daily basis. This added difficulty in their life indirectly prevents them from reaching out to a professional in the early course of their disease.

After 2 weeks she returned and was happy that she was free of her symptoms. She thanked me and handed me a bag of fruit. Before I could say anything, she said, “Now do not give me money for this. This deed is out of pure love, which money cannot buy. You gave me something I never thought would happen: a peaceful night’s sleep. So now it’s my turn to return the favor. This too is professional ethics.”

Through all my experience as a doctor, I had gone through training on so many aspects of what professional ethics means. I have seen so many educated people, even my own peers, stumble with professionalism. Here, high-quality professionalism came from a normal person with very minimal education. She did not lower her standards for anyone or anything. The way she treated herself set her apart and clearly dictated the terms on how she wanted to be treated by others.

There is substantial literature on scabies and its impact on socioeconomic aspects, but to see how a small mite can push a human to an unceasing distress reminds us of nature’s ethics. As a professional I deal with the daily portion of dilemmas and find myself greatly impacted by this one woman. For the emerging generations with colossal dreams who will also have to deal with many dilemmas, I hope they can learn from this uneducated woman that, regardless of whatever you have learned, integrity and ethics set you apart.

